# Medical and Physical Intelligence

**Published:** 1805-09-01

**Authors:** 


### MEDICAL AND PHYSICAL

### INTELLIGENCE.

[ FOREIGN AND DOMESTIC. ]

*A Memorial of the Medical Committee, addressed to the Inhabitants of the City of Norwich, and of the County of Norfolk, in support of a plan for the extinction of the small-pox by a general inoculation for the cow-pox.*

In this age of mutual charity and benevolence, any address, from any class of men, in behalf of their fellow creatures, daily suffering from a dangerous and loathsome disease, might be thought unnecessary by superficial observers. It is, however, a painful reflection, that this is not the case: for the constant existence of the casual Small-Pox, in the united kingdom of Great Britain and Ireland, and its increase, during the last few months, in this city and county, afford an ample proof, that it is an evil of great extent, tent, not only requiring the serious attention of an enlightened legislature, but more particularly appealing to the judgment and feeling of such persons, the object of whose duty and professional employment is the prevention and removal of disease. In speaking of the nature of casual Small-Pox, it may with truth be observed, that it has been the scourge of human nature for many centuries, and that it has surpassed every other disease in virulence and devastation: even the plague itself, whose very name spreads terror through the world, yielding to it in the mortality and loss of human life which it occasions, although armed with the power of attacking the same individual more than once. The reason of this greater mortality from small-pox than plague, or any other disease, will readily occur to the thinking mind; the ravages of the plague are softened and restricted by season, and receive limitation from soil and climate; but no controul of this nature arrests the progress of small-pox, its baneful influence existing at all times and seasons, and visiting every soil and every climate: so that it may be emphatically termed, a general and perpetual plague. That this character, however dreadful, of small-pox is true, we need only recur to the bills of mortality, where an account of the deaths arising from both diseases is given, and from them it appears, that the deaths from plague within the London bills, during a century, viz. from 1601 to 1701 inclusive, were 188,571, but from 1701 to 1801 inclusive, it appears, that in a century, at least 250,000 persons have perished of this fatal disease, the average of deaths being, in the same bills, considerably more than 2000 a year. And we may with safety presume, that a similar ratio of the mortality of small-pox and plague obtained, during the same period, in those parts of the kingdom, unnoticed by the London bills.

But this statement taken from records, warped by no theory, and subservient to no speculation, shews only the immediate or direct mortality of small-pox, and affords us no clue to estimate the morbid effects, which it exerts upon the human frame, in producing death more remotely, or by the intervention of other diseases; such as its power of forming scrophula, or calling into action its latent seeds, the most fertile cause of consumption, a disease too fatal, and too general, in the British isles. These indirect effects of small-pox, in producing death, or entailing upon mankind other diseases, as permanent weakness of body, diminished strength of limb, loss or defect of sense, as blindness and deafness, can only be accurately known to medical men, whose daily practice brings them acquainted with the victims of small-pox, whether from its direct or remote influence. It is not to be wondered at, then, that the generous and enlightened should, for a long period, have been employed in inventing means adequate to the resistance and suppression of so great an evil to society as small-pox; and we find, so long ago as the beginning of the last century, about 1722, a method of communicating this disease, disease, by inoculation, was brought to this kingdom, and which certainly disarmed it of its danger, in a very great degree. Humanity received this discovery with eagerness, and reason augured from it the greatest good; and it was thought, that human nature would no longer be the victim of so malignant a disease. It appears that these hopes were too sanguine, for history informs us that this mode of giving the disease by inoculation, was at first partially received, and of course but slowly practised; and that after a lapse of a few years, it became more general among the higher orders of society, and institutions were formed in the metropolis, and in other parts of the kingdom, for the gratuitous inoculation of the lower.—It was hoped, by these salutary proceedings, not only, that great restraint would be put upon the progress of a disease, destroying in the first fifteen years of life, more than the combined power of all other diseases, but that ultimately the extinction of it would be effected. This goodly prospect, so far from being realized, has been wholly darkened by some melancholy facts, which inquiry has established; and from which it appears that more deaths have taken place since the practice of inoculation than before; an effect which is only to be ascribed to the practice of inoculation among the higher orders; keeping up the casual small-pox among the lower, in a great degree;—for compared with casual small-pox, the inoculated may be said to be almost free from danger; the proportion of deaths from the inoculated being only one in three hundred, whereas it appears, from the casual small pox, at a moderate calculation, about one in six falls a victim to the disease. It was soon discovered, that, to remedy these mischievous effects of partial inoculation, and to protect human nature from the casual small-pox, it was necessary to extend the practice of inoculation generally, or, more strictly speaking, universally — and it has been the aim of the first medical and political characters to inculcate the necessity of substituting universally the inoculated for the natural small-pox.—It might have been supposed that a measure dictated by reason and common sense, and by which the fatality of small-pox might have been so greatly abridged, would have met with little difficulty in the execution--but these humane exertions have failed altogether, as within the last few years the mortality from small-pox has been annually increasing; for it appears that in the first thirty years of the eighteenth century, before the effects of inoculation could be shewn, in every 1000 deaths, the proportion arising from small pox amounted only to 74, but in the last thirty years of that century, the deaths from small-pox amounted to 95 in 1000. It is our opinion, that this failure, in extending generally the inoculation of small-pox, is not so much to be ascribed to the prejudices of the poor, as to a supineness of character respecting disease, or insensibility to their own safety, the necessary atributes of poverty, or of minds whose exertion is unceasingly directed to procuring daily sustenance for themselves and families. This is evinced by the readiness with which the poor, when called upon, submit to parochial inoculation.

But, whatever melancholy impressions these failures may have made upon the minds of medical men or others, they have been greatly removed, by a new æra in medicine, commencing with the discovery of cow-pox inoculation, by Dr. Jenner, as a preventive of small-pox. A discovery which should be received and rewarded, not only by individual, but by national, gratitude. In this discovery, as far as the experiment has been made, there is reason to believe, that he has afforded mankind a complete antidote, a certain protection against small-pox, and has, probably, given them the means of extinguishing the small-pox altogether. In giving our suffrage to this discovery, and to the application of it, it is our wish to impress upon the minds of the inhabitants of this city and county, our thorough conviction of its safety, and of its efficacy, when duly employed in preventing, small-pox—that we consider its action upon the human frame the most mild and innocent, never proving fatal, and requiring no assistance from internal medicine—and lastly, that it is not contagious—and on these accounts, it requires no suspension of intercourse or industry, among the different members of society. And as far as our experience has gone, the cow-pox never calls forth or is followed by any other disease, such as the evil, general disability of body, or loss or imperfection of any of the senses. In addition to these facts, we wish further to remark, that in our experience during the last five or six years, we have never witnessed an instance of a person receiving the small-pox, after having been duly and attentively subjected by inoculation to the cow-pox; and we are of opinion, that the numbers which have been inoculated for it, in the united kingdom of Great Britain and Ireland, (not to mention the other parts of the world) for the last five or six years, afford an ample testimony, an incontrovertible experiment, of its preventive power; not knowing any analogy of action, or law of the animal œconomy, by which it is rendered doubtful, that the preventive effects of cow-pox, upon the human body, after being exerted, for four or five years, should not continue to be exerted through life. And that this is the fact is proved by many cases on record, of persons receiving the disease from the cow, in early years, and never afterwards, through a long life, taking the small pox, although exposed to its action. With this conviction, we are called upon as friends of science and humanity, to recommend to the inhabitants of this city and its hamlets, the adoption of the general cow-pox inoculation, proposed at the General Meeting of the inhabitants of this city, on Monday last, July 29th.

That this recommendation may receive every assistance that a candid and impartial inquiry can give it, we shall briefly examine the force of the objections brought against cow-pox inoculation, and draw a parallel between its effects and the effects of inoculated small-pox upon the human body.—It is admitted on all hands, that the the cow-pox is never fatal, whereas in the inoculated small-pox, one in 300 perishes; a circumstance of no trifling consideration. It is asserted that cow-pox is not, universally, a security against small-pox, there being instances alleged of persons taking the small-pox, after having been inoculated for the cow-pox. To give this argument its full force, let us admit all the alleged cases to be true, and then proceed to ascertain the proportion they bear to the whole number of persons who have been inoculated for the cow-pox.—From this inquiry it appears, that of 250,000 persons, who have been inoculated for the cow-pox, only 50 persons have been alleged to have suffered from subsequent small-pox; thus, even in this view, the cow-pox is highly to be preferred to the small-pox, as, from this estimate, *only one person in* 5000 *is liable to small-pox, whereas in inoculated small-pox, it is admitted, that one person in* 300 *perishes*.—But a more minute investigation has shewn, that of these fifty alleged cases, only ten have been substantiated by evidence admissible and adequate; and that it is probable, among these ten cases, some deception or mistake may have taken place, on the same grounds, as in some of the asserted cases of small-pox subsequent to small-pox, and in which the chicken-pox has been taken for small-pox.—Admitting, however, these ten cases to be established, the conclusion from such admission strongly proves the superior advantages of cow-pox inoculation; as in that case, instead of one person in 5000, only one person in 25000 would be liable to small-pox. And farther supposing in the 250,000 persons inoculated for the cow-pox, that ten of them (as asserted) should be liable to small-pox, and should actually take it, and in the casual way, and that of these ten one in five should die, which is a greater proportion than really obtains, it would then appear, that of 250,000 persons inoculated for the cow-pox, only two persons would have died, and those from subsequent small-pox; whereas, the deaths from the same number of persons (250,000) inoculated for small-pox (taking the received proportion of one in 300), would be about 834.—Thus it is proved, that the fatality of small-pox inoculation, compared with that of persons taking the small-pox in the casual way, subsequent to the cow-pox, is, as near as may be, 834 to 2; a fact at once strongly exhibiting the superior advantages and mildness of the cow-pox, when compared with small-pox.

With this fact we shall conclude our remarks, trusting, that enough has been said to incite the inhabitants of this city, to adopt the proposed plan of general cow-pox inoculation, and that the poorer classes of society will, with gratitude, listen to these friendly councils, and practice a plan so necessary to their safety: —and that the Court of Guardians of the Poor, the Clergy, and leading inhabitants of this city, will assist and promote measures so beneficent and salutary, not only by their influence but by their example, by discouraging, on the one hand, the pernicious practice of inoculating for the small-pox, and, on the other, advancing, by their utmost endeavour, the adoption of the cow-pox.—

And

And for the same reason must we appeal to the Ministers, to the parish officers and leading men of the several parishes in this county, for their co-operation, in discouraging the inoculation of the small-pox, and in adopting that for the cow-pox; knowing as we do, that the characters mentioned, have with parental attention, and from the best motives, encouraged every few years, in their several parishes, a general inoculation for the small-pox, a practice eventually highly pernicious, as, at the same time that it gives security to the parishes inoculated, it carries danger and death to the adjoining parishes, in which inoculation has not taken place.

And as experience has shewn the little progress which has been made at all times, by the small-pox inoculation, among the poor, when left to themselves, and unsolicited to apply it in practice; a fact established beyond a doubt, by the reflection that only the small number of 25,000 persons have been inoculated during the last forty years of the last century, at the Inoculating Hospital, in London, a very extensive institution;—we therefore recommend that the children of the poor, or other persons, who have not had either the small-pox or the cow-pox, be once or twice a year, or occasionally, inoculated at their own houses; a measure necessary to render permanent the good effects of a general cow-pox inoculation, in extinguishing the small-pox. We think, moreover, in this universal cow-pox inoculation, it will be prudent for a time, as there is frequently no visible constitutional disturbance of the system, denoting its efficient agency, that the part inoculated should be submitted to the inspection of some medical men, or to some person conversant in the practice; by these means mistakes may be prevented, prejudicial to the individual, and to the extension of the cow-pox inoculation; and should hereafter any solitary case of small-pox arise, from any secret or unknown source, it is hoped that all intercourse will be stopped immediately, between persons so affected, and such as are liable to the disease.

That these hopes are not too sanguine, the present state of Vienna, the metropolis of Germany, evinces; for in that city, containing a population of 254,000, after a general cow-pox inoculation, only two persons have died of small-pox during the year 1804.

By enforcing these measures, we shall soon see the united kingdom of Great Britain and Ireland rival the other states of Europe, not only in religion, science, and morals, but in consulting the safety, and securing the lives, of our fellow creatures, by the extinction of the small-pox—and that our commerce will no longer be charged with carrying the seeds of death and destruction to distant quarters of the globe, at the same time that it conveys the products of our industry, and the arts of civilized life.

(SIGNED)

| | | |
|---|---|---|
| RICH. LUBBOCK | WM. BACK | WM. FELL RAND |
| WARNER WRIGHT | JAMES KEYMER | C. W. STARKEY |
| EDWARD RIGBY | JAMES ROBINSON | SAM. S. DEACON. |
| P. M. MARTINEAU | WM. DALRYMPLE | |

*Extract of a Letter from Dr. Benjamin Waterhouse to the Editors, dated March* 13, 1805.

Dr. De Carro sent me some vaccine matter on an ivory lancet enclosed in a wooden case; the letter which accompanied it was dated Vienna, October 28, 1803. I made use of it November 27, 1804; when it communicated the genuine pustule and disease. Does the history of vaccination afford another instance of the virus communicating the true disorder after being taken from the subject thirteen months?

---

The Plates for Mr. Saunders's Work on the Structure of the Ear, are in the hands of Mr. Heath, and it is expected to be ready for publication early in October.

---

A new volume of the valuable Transactions of the London Medical Society is announced as ready for publication.

---

Mr. James Briggs will shortly lay before the public, Practical Observations on the principal Diseases of the Eyes, illustrated by Cases, translated from the Italian of Antonio Scarpa.

---

Mr. Donovan is printing an Epitome of the Natural History of the Insects of New Holland, New Zealand, New Guinea, Otaheite, and other Islands in the Indian, Southern, and Pacific Oceans; including the Figures and Descriptions of one hundred and fifty-two Species of the most splendid, beautiful, and interesting Insects hitherto discovered in those Coun tries

---

The second volume of Bell's Surgery, containing operations of surgery, may be expected in a short time.

---

University of Glasgow.

The Medical Lectures in the University of Glasgow, will begin on Tuesday the 5th of November, at the following hours:

Dietetics, Materia Medica and Pharmacy, by Dr. Millar, at ten o'clock forenoon.

Midwifery, by Mr. Towers, at eleven.

Theory and Practice of Physic, Dr. Freer, at twelve.

Anatomy and Surgery, by Dr. Jeffray, at two o'clock afternoon.

Chemistry and Chemical Pharmacy, by Dr. Cleghorn, at seven.

Clinical Lectures on the Cases of Patients in the Royal Infirmary, by Dr. Cleghorn and Dr. Freer. The First Lecture by Dr. Cleghorn, on Tuesday evening the 12th of November, at six o'clock.

Dr. Brown will begin his Lectures on Botany, about the beginning of May next.

*Regulations*

*Regulations enacted by the Senate of the University of Glasgow, respecting Degrees of Medicine.*

1. That before any person can be allowed to be a Candidate for a Degree in Medicine in this University, he shall appear personally before the Senate, and lay before them evidence, that during the space of three years, or sessions of six months each, he has regularly attended in some University or Universities, or in some medical school or schools of reputation, the following medical classes, viz. Anatomy and Surgery, Chemistry and Pharmacy, the Theory and the Practice of Physic, Materia Medica, and Botany.

2. That he shall bring forward evidence, that during one year at least, he has attended Medical Lectures in this University.

3. That the Candidate shall undergo three separate examinations in private, by the Medical Professors of the University, and write a Commentary on an Aphorism of Hippocrates, and another on a case of disease propounded to him by the said Examiners. The first examination shall be on Anatomy and Physiology; the second, on the Theory and Practice of Physic; and the third, on Chemistry, Materia Medica, Pharmacy and Botany.

4. That the Examiners shall report to the Senate, their opinion respecting the medical knowledge of the Candidate; and if their report be favourable, his name as a Candidate for a Degree, shall be entered in the minutes of the Senate, and a day fixed, when the Candidate shall read his Commentaries on the Aphorism and Case, and answer such questions on the several branches of medical science, as shall be put to him by the Examiners, in presence of the Senate. If the Senate be of opinion, that the Candidate has shewn himself worthy of a Degree, it shall be conferred in presence of the Senate, by the Vice Chancellor, provided the Candidate has not published a Thesis; which he may, or may not do, according to his own option; but if he has published a Thesis, he must defend it, and the Degree must be conferred in the Comitia.

5. That the whole of the examinations shall be carried on, and the Commentaries on the Aphorism and Case must be written in the Latin language.

---

### Medical Lectures.

We recur with satisfaction to our annual task of announcing the various Medical, Surgical, and Scientific Lectures delivered during the Winter Season in this Metropolis. The experience of the various Lecturers, their extensive practice in this populous City, and the numerous cases always furnished of every disease by our great Hospitals, necessarily render London THE FIRST SCHOOL OF PRACTICAL MEDICINE IN THE WORLD. We are happy to find that this truth begins to be properly understood, and that the classes of the various Lecturers are every year greatly increased in number, not only in native Students, but but in Foreigners from every University in Europe and America, so as to make a total number of six or seven hundred in every Season.

The following Courses of Lectures will be delivered at the Medical Theatre, St. Bartholomew's Hospital, during the ensuing winter:—On the Theory and Practice of Medicine, by Dr. ROBERTS and Dr. POWELL. On Anatomy and Physiology, by Mr. ABERNETHY. On the Theory and Practice of Surgery, by Mr. ABERNETHY. On comparative Anatomy and Physiology, by Mr. MACARTNEY. On Chemistry, by Dr. EDWARDS. On the Materia Medica, by Dr. POWELL. On Midwifery and the Diseases of Women and Children, by Dr. THYNNE. The Anatomical Demonstrations and Practical Anatomy, by Mr. LAWRENCE. The Anatomical Lectures will begin on Tuesday, October 1, and the other Lectures on the succeeding days of the same week. Further particulars may be learned by applying to Mr. Nicholson, at the apothecary's shop, St. Bartholomew's Hospital.

---

The Winter Course of Lectures given at the adjoining Hospitals of ST. THOMAS's and GUY's will commence in the following order.—At St. Thomas's:—Anatomy and the Operations of Surgery, by Mr. CLINE and Mr. ASTLEY COOPER, on Tuesday, October 1, at one o'clock. Principles and Practice of Surgery, by Mr. COOPER (illustrated by select Cases under his care in Guy's Hospital), on Monday, October 7, at eight in the evening. —At Guy's Hospital:—Practice of Medicine, by Dr. BABINGTON and Dr. CURRY, Wednesday, October 2, at ten in the morning. Principles and Practice of Chemistry, by Dr. BABINGTON and Mr. ALLEN, on Thursday, October 3, at ten in the morning. Midwifery, and Diseases of Women and Children, by Dr. HAIGHTON, on Friday, October 4, at eight in the morning. Pathology, Therapeutics, and Materia Medica, by Dr. CURRY, on Friday, October 4, at eight in the evening. Physiology, or Laws of the Animal Economy, by Dr. HAIGHTON, on Monday, October, 7, at a quarter before seven in the evening. Experimental Philosophy, by Mr. ALLEN (Lecturer at the Royal Institution), on Tuesday, October 8, at half past six in the afternoon. Clinical Lectures on select Medical Cases, from November till May, by Dr. BABINGTON, Dr. CURRY and Dr MARCET. Besides these, a Course of Lectures will be given on Veterinary Medicine, by Mr. COLEMAN, Professor at the Veterinary College. And one on the Structure and Diseases of the Teeth, by Mr. FOX, Surgeon-Dentist. These several Lectures are so arranged that no two of them interfere in the hours of attendance; and the whole is calculated to form a complete Course of Medical and Chirurgical Instruction. Terms and other particulars may be learnt by applying to Mr. STOCKER, apothecary to Guy's Hospital; who is also empowered to enter gentlemen as pupils to such of the Lectures as are delivered at Guy's.

---

St. George's Hospital.—The first Monday in October next will commence a Course of Lectures on Physic and Chemistry, at the Laboratory in Whitcomb Street, at the usual morning-hours, viz. on the Therapeutics at a quarter before eight; on the Practice of Physic at half after eight; and on Chemistry at a quarter after nine o'clock; by George Pearson, M. D. F. R. S. of the College of Physicians, and Senior Physician to St. George's Hospital, &c. &c. These Lectures are delivered every morning, except on Saturdays, when a Clinical Lecture is given, on the cases of patients in St. George's Hospital. Proposals may be had at St. George's Hospital, and at No. 14, Leicester Square.

---

Mr. Headington and Mr. Frampton will commence their Autumnal Course of Lectures at the Theatre of the London Hospital, on Anatomy, Physiology, and the Principles and Operations of Surgery, on the 1st of October, at two o'clock. The Anatomical Demonstrations and Dissection by Mr. Armiger. —Dr. Dennison will Lecture at the same place on the Theory and Practice of Midwifery.

---

The established plan of instruction for the benefit of pupils attending the Westminster Hospital, will be pursued throughout the ensuing winter, under the direction of Mr. Lynn and Mr. Carlisle. Further particulars may be had at the Hospital in James Street.

---

Dr. Badham's Autumnal Course of Lectures on the Institutions and Practice of Medicine, Chemistry, &c. will be commenced on October the 15th, at his Laboratory, in Clifford Street, at eight o'clock, and continued at the same hour. To this and to the succeeding course of lectures, those gentlemen who had become perpetual pupils to Dr. Crichton, will be admitted. A prospectus may be had.

---

Dr. Batty's usual Course of Lectures on the Theory and Practice of Midwifery, and on the Diseases of Women and Children, will commence on Monday, October 7, at his house in Great Marlborough Street, at half past ten o'clock.

---

Mr. Blair's Lectures on the Natural History of Man (for the information of scientific and professional gentlemen, amateurs of natual history, students in the liberal and fine arts, &c.) will recommence on the 28th of January, at the Bloomsbury Dispensary, Great Russell Street; to be continued every succeeding Tuesday and Friday evening, at eight o'clock precisely, until the termination of the course, which will consist of about twenty lectures.

---

Dr. Bradley will commence his Autumnal Course of Lectures on the Theory and Practice of Medicine, in the second week of October.

Mr. Brookes will commence his Autumnal Course of Lectures at the Theatre of Anatomy, Blenheim-street, Great Marlborough-street, on Anatomy, Physiology, and Surgery, on Tuesday, Oct. 2, at two o'clock. Spacious apartments, thoroughly ventilated, and replete with every convenience, will be open in the morning till two, for the purposes of dissecting and injecting, where Mr. Brookes attends. An extensive museum appertains to the Theatre.

---

Mr. Carpue will commence his Anatomical Lectures on the 30th of September. The Dissecting Room will be open from eight o'clock in the morning till five in the afternoon. Three courses are given in the year. Further particulars may be had of Mr. Carpue, at his house, No. 50, Dean Street, Soho.

---

Mr. Chevalier, surgeon extraordinary to the Prince of Wales, and surgeon to the Westminster General Dispensary, will begin his Winter Course of Lectures on the Principles and Operations of Surgery, on Monday the 7th of October, at seven o'clock in the evening, at his house in South Audley Street, Grosvenor Square, where printed particulars may be had.

---

Dr. Clarke will begin his usual Course of Lectures on the Theory and Practice of Midwifery, and the Diseases of Women and Children, on Friday the 4th of October, at the Lecture Room, No. 10, Upper John Street, Golden Square. For the convenience of gentlemen attending the different hospitals, these lectures will be given from a quarter past ten to a quarter past eleven in the morning. Particulars may be known by applying to Dr. Clarke, Burlington Street, or to Mr. Clarke, at the Lecture Room.

---

Mr. Milburne's Physiological Lectures, illustrated by Anatomical preparations, casts, drawings, &c. &c. will recommence the first Monday evening in October; to be continued every succeeding Monday evening, as eight o'clock precisely.

---

Dr. Reid's First Winter Course of Lectures, considerably enlarged, will commence early in October, and will be delivered in some part of the city. Particulars may be learned from Dr. Reid, Grenville Street, Brunswick Square.

---

Mr. John Taunton, Member of the Royal College of Surgeons in London, Surgeon to the City Dispensary, &c. will commence his first Winter Course of Lectures on Anatomy, Physiology, Pathology, and Surgery, in October next, at the Theatre of Anatomy. An ample field for professional instruction will be afforded by the privilege which the pupils may enjoy, by attending the clinical practice of both the City and Finsbury Dispensaries. Lectures will be delivered on the Theory and Practice of Medicine, by Dr. Reid; and on Midwifery, including the Diseases of Women men and Children, by Dr. SQUIRE. Further particulars may be known on application to Mr. Taunton, No. 10, Pater-noster Row, Cheapside.

Mr. THELWALL, whose intention of commencing a Course of public and private Instructions in Elocution, in the metropolis, during the ensuing winter, we have already noticed, has, in the mean time, opened a seminary at Brownlow Hill, Liverpool, for the Relief of persons afflicted with *Impediments of Speech*, and the instruction of foreigners desirous of being initiated in the idiom and pronunciation of the English language.

At the Theatre of Anatomy, in Great Windmill Street, Mr. WILSON's Lectures on Anatomy, Physiology, Pathology, and Surgery, will begin on Tuesday, the 1st of October. Two courses of lectures are read during the winter and spring seasons.—In the first course is explained the Structure of every part of the Human Body, so as to exhibit a complete view of its Anatomy, as far as it has been hitherto investigated; to which are added, its Physiology and Pathology. In the second course, the Structure of the Human Body is again explained; after which follow Lectures on the Operations of Surgery; and the course concludes with the Anatomy of the *Gravid Uterus*. A Lecture is given daily from two till four o'clock. Practical Anatomy in the mornings as usual. A plan and terms of the course may be had at the Theatre.

---

INDEX TO THE FIRST SERIES OF THE MEDICAL AND PHYSICAL JOURNAL.

